# Diagnostic test accuracy of point-of-care procalcitonin to diagnose serious bacterial infections in children

**DOI:** 10.1186/s12887-020-02385-2

**Published:** 2020-10-21

**Authors:** Thomas Waterfield, Julie-Ann Maney, Mark D Lyttle, James P McKenna, Damian Roland, Michael Corr, Bethany Patenall, Michael D Shields, Kerry Woolfall, Derek Fairley, On behalf of Paediatric Emergency Research in the UK and Ireland (PERUKI)

**Affiliations:** 1 Emergency Department, Royal Belfast Hospital for Sick Children, Belfast, UK; 2 Centre for Experimental Medicine, Wellcome Wolfson Institute of Experimental Medicine, Queen’s University Belfast, Belfast, UK; 3 Emergency Department, Bristol Royal Hospital for Children, Bristol, UK; 4 Faculty of Health and Applied Sciences, University of the West of England, Bristol, UK; 5 Department of Microbiology, Belfast Health and Social Care Trust, Belfast, UK; 6 SAPPHIRE Group, Health Sciences, Leicester University, Leicester, UK; 7 Children’s Emergency Department, Leicester Royal Infirmary, Leicester, UK; 8 Belfast Health and Social Care Trust, Belfast, UK; 9 Department of Chemistry, University of Bath, Bath, UK; 10 Institute of Population Health and Society, University of Liverpool, Liverpool, UK

**Keywords:** Procalcitonin, infection, paediatrics, bacterial infection, biomarkers

## Abstract

**Background:**

The National Institute for Health and Care Excellence (NICE) have called for research into the role of biomarkers, and specifically procalcitonin (PCT), for the early diagnosis of serious bacterial infections (SBI) in children. The aim of this study was to compare the diagnostic test accuracy of C-reactive protein (CRP) and PCT for the diagnosis of SBI in children.

**Methods:**

Data was collected prospectively from four UK emergency departments (ED) between November 2017 and June 2019. Consecutive children under 18 years of age with fever and features of possible sepsis and/or meningitis were eligible for inclusion. The index tests were PCT and CRP and the reference standard was the confirmation of SBI.

**Results:**

213 children were included in the final analysis. 116 participants (54.5%) were male, and the median age was 2 years, 9 months. Parenteral antibiotics were given to 100 (46.9%), three (1.4%) were admitted to a paediatric intensive care unit and there were no deaths. There were ten (4.7%) confirmed SBI. The area under the curve for PCT and CRP for the detection of SBI was identical at 0.70.

**Conclusions:**

There was no difference in the performance of PCT and CRP for the recognition of SBI in this cohort.

**Trial registration:**

Registered at https://www.clinicaltrials.gov (trial registration: NCT03378258) on the 19th of December 2017.

## Background

Serious bacterial infections (SBI) can be difficult to distinguish from many self-limiting benign viral infections affecting children, especially during the prodrome. When the diagnosis is unclear, clinicians may use biomarkers, such as C-reactive protein (CRP) and procalcitonin (PCT), to aid clinical decision-making [1]. Procalcitonin is the precursor for calcitonin and is produced by parafollicular cells [2, 3]. It is a 116-amino acid protein that has roles in calcium metabolism [4]. PCT is elevated during infection and typically rises within two hours of the onset of a bacterial infection reaching a peak at 24 to 36 hours [4]. Procalcitonin levels are attenuated by the presence of interferon gamma that is typically released during viral infections leading to suggestions that PCT may have uses in distinguishing viral from bacterial infections [4].

The existing literature regarding the test accuracy of PCT in children is favourable with at least five meta-analyses demonstrating that PCT is accurate when used to diagnosis SBI across a range of paediatric settings [5–9]. The two most commonly used PCT cut-offs are 0.5 ng/ml and 2.0 ng/ml [5–9]. The lower cut-off of 0.5 ng/ml typically provides an approximate 80% sensitivity for the identification of SBI whereas the higher cut-off of 2.0 ng/ml typically provides a specificity of around 90% [5–9]. Two of the five meta-analyses compared PCT to CRP with both studies reporting that PCT was more accurate than CRP for the diagnosis of SBI in children [8, 9]. PCT has also been shown to be particularly useful in the assessment of febrile young infants under 90 days of age [10–12].

As technology evolves, there is increasing interest in the use of point-of-care (POC) biomarkers for the early recognition of SBI. There have been a number of studies exploring the use of POC CRP testing to identify SBI at presentation to healthcare [13, 14]. These studies have reported that the use of POC CRP can help to risk stratify children at triage/initial assessment [13]. Procalcitonin testing may however, represent the ideal POC test for detecting SBI in children due to its greater test accuracy.

The purpose of this sub-analysis of the Petechiae in Children (PiC) study was to report the diagnostic test accuracy of POC PCT for the diagnosis of SBI in children presenting to the Emergency Department (ED).

## Methods

The PiC study was a mixed method prospective, multicentre cohort study, for which the full protocol is available as an open access publication [15]. The PiC study was designed and reported in line with the Standards for Reporting Diagnostic accuracy studies (STARD) statement [16].

### Participants

Consecutive children under 18 years of age (initially limited to 14 years but extended to 18 years following an amendment) attending Emergency Departments (ED) with reported or recorded fever (≥ 38 °C) and features of meningococcal infection, sepsis, or meningitis with blood available for PCT testing were eligible for inclusion. Features of meningococcal infection included a non-blanching rash or a global assessment of likely sepsis/meningitis based on National Institute for Health and Care Excellence (NICE) guidance CG120 [17].

Children with pre-existing conditions predisposing to non-blanching rashes were excluded as were participants who were screened after admission when reference tests had already been performed. Participants for this sub-analysis were enrolled between the 9th of November 2017 and the 30th of June 2019 at four EDs in the UK (recruitment by site can be found in Table 1).

**Table 1:** Summary for the 213 study participants (n and (%) unless otherwise stated)

Characteristic	
Recruitment by site	
Royal Belfast Hospital for Sick Children (Tertiary Children’s Hospital)	198
Salisbury District Hospital (District General Hospital)	11
Royal Lancaster Infirmary (District General Hospital)	3
Furness General Hospital (District General Hospital)	1
Demographic Data	
Age months; median (range)	33 (1–167)
Male sex	116 (54.5)
Vaccination status	
Vaccinations up-to-date	208(97.8)
Index Tests	
PCT	213(100)
C-reactive protein	213(100)
Reference Standard Tests Performed	
At least one reference test performed	209(98.1)
Blood Culture/PCR	208(97.7)
Urine Microscopy/Culture	46(21.6)
Stool Culture	7(3.2)
Chest Radiograph	5(2.3)
CSF Microscopy/Culture/PCR	2(0.9)
Histology	0(0.0)
Outcomes	
Received parenteral antibiotics at first presentation	100 (46.9)
Admitted to PICU	3(1.4)
Unplanned re-attendance	3(1.4)
Deaths	0(0.0)
Serious infections	10(4.7)
• Septicaemia	4(1.9)
• Bacterial Meningitis	1(0.5)
• Appendicitis	0(0.0)
• Pneumonia	0(0.0)
• Osteomyelitis	0(0.0)
• Cellulitis	3(1.4)
• Bacterial gastroenteritis	0(0.0)
• Urinary tract infection	2(0.9)

### Test methods

The PCT index test was performed using the commercially available BRAHMS IB10 PCT test, on 0.5 ml of whole blood collected in Lithium Heparin paediatric blood bottles during routine phlebotomy. Samples were tested as soon as possible after collection using the Samsung LABEGO IB10 analyser, housed in the ED. The stepwise procedure involved pipetting 0.5 ml of the sample into the test disc before loading this onto the analyser and pressing run. The total run time for each sample was 20 minutes. In all instances the index test was performed prior to the reference standard results being available. The PCT result was not made available for use in the child’s clinical care. The CRP testing was performed in accredited NHS laboratories as part of the child’s routine investigations.

### Reference standard

The reference standard was the diagnosis of a serious bacterial infection (SBI), the criteria for which are detailed below based on previously published definitions [5–10]. The reference standard tests were performed blinded to the result of the index tests except for clinical diagnoses such as cellulitis where an independent reference test does not exist.

- Septicaemia (including bacteraemia): pathogenic bacteria isolated from blood culture or polymerase chain reaction (PCR).
- Bacterial Meningitis: Identification of bacteria in cerebrospinal fluid using culture or PCR.
- Appendicitis: confirmed on histology.
- Pneumonia: infiltrate on chest X-ray confirmed by consultant radiologist.
- Osteomyelitis: pathogens from bone aspirate, or MRI or bone scan suggestive of osteomyelitis.
- Cellulitis: acute suppurative inflammation of subcutaneous tissues.
- Bacterial gastroenteritis: pathogen isolated from stool culture.
- Complicated urinary tract infection: >10^5^/mL pathogens of a single species isolated from urine culture and systemic effects such as fever.

### Follow up

Researchers at each site checked attendance records seven days after enrolment to monitor for any unplanned re-attendances by study participants. Where this occurred it was logged along with any change of diagnosis.

### Analysis

The study population was described in terms of demographic characteristics. Simple descriptive statistics (total number and proportion) were used to describe vaccination status, parenteral antibiotic use, admission to intensive care units, and survival. The diagnostic accuracy of PCT and CRP at a range of cut-points was reported as sensitivity, specificity, negative predictive value (NPV), positive predictive value (PPV) and negative/positive likelihood ratios (LHR), with 95% confidence intervals (CI). The area under the curve (AUC) was calculated for both PCT and CRP. Patients where the index test PCT was not performed were excluded from analysis.

### Consent Model

Due to the potentially life-threatening nature of SBI a research without prior consent (RWPC) model was used as described in detail elsewhere [18]. All participants were invited to provide written consent at the earliest appropriate opportunity once the child’s clinical condition had stabilised (typically within 24 hours of enrolment). All participants were free to decline consent without suffering any detriment.

### Data Management

Study data were collected and managed using REDCap (Research Electronic Data Capture) [19]. Copies of the case report forms are available in the supplementary material.

### Public and patient involvement (PPI)

PPI was integral within the PiC study from its outset. The PPI group assisted in its design including the protocol, study information, and RWPC methodology.

### Office for Research Ethics Committees (OREC) and local Research Governance

The study was approved by both the Northern Ireland OREC (Project ID 224,660, OREC ID 17/NI/0169) and by the Belfast Health and Social Care Trust Research Governance.

### Study Registration

The PiC study was registered at https://www.clinicaltrials.gov (trial registration: NCT03378258) on the 19th of December 2017.

## Results

**Fig. 1:**
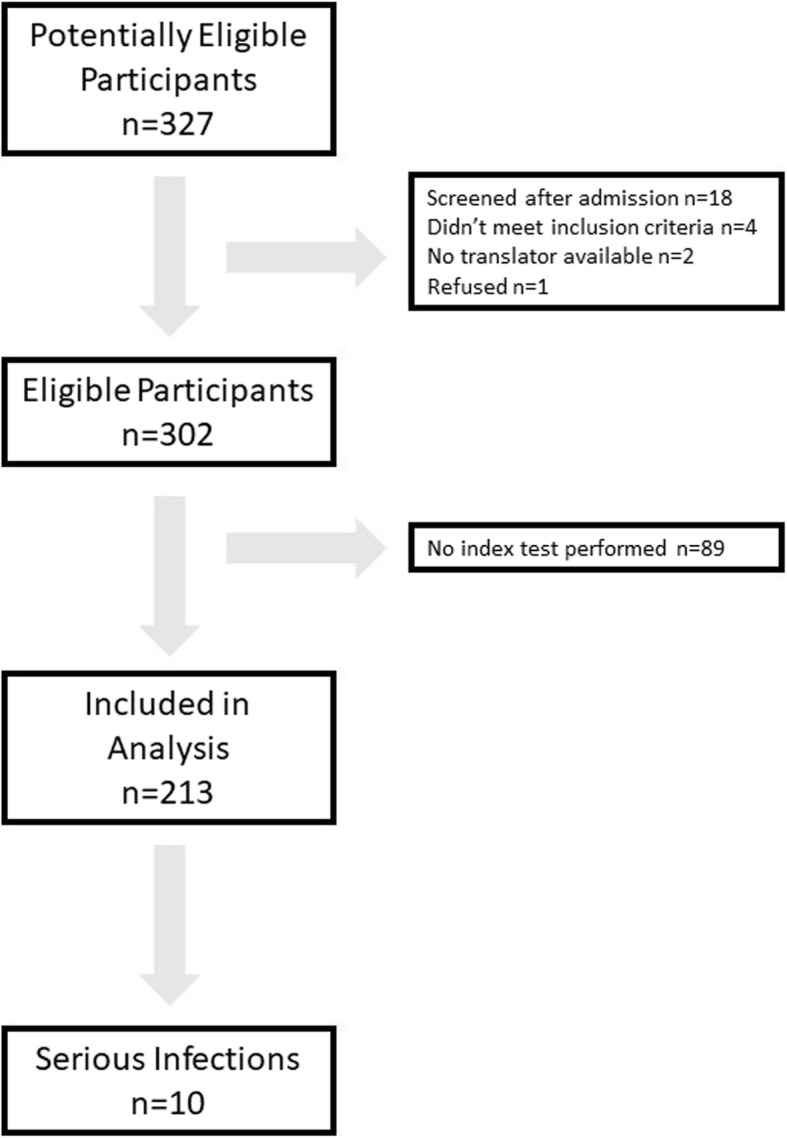
Flow of participants through the study

A total of 327 consecutive children were screened for eligibility across the four sites, of which 25 were ineligible and 89 did not have sufficient blood available for PCT testing (Fig. 1). Amongst the 89 with insufficient blood available for PCT testing there were four serious bacterial infections (4.5%). The SBI comprised of one case of septicaemia, one urinary tract infection and two children with pneumonia.

A total of 213 children were included in the final analysis. Reference standard testing was performed in all but four participants; all four were diagnosed with a viral illness, were discharged, and did not re-attend. 116 participants (54.5%) were male, and the median age was 2 years, 9 months (range 1 month − 13 years, 11 months), with 166 (77.9%) aged five years or younger. A total of 208 (97.8%) were appropriately vaccinated for age according to the UK vaccination schedule. Parenteral antibiotics were given to 100 (46.9%), three (1.4%) were admitted to a paediatric intensive care unit (PICU), and there were no deaths. There were ten (4.7%) confirmed SBI (Table 1). The AUC for PCT and CRP for the detection of SBI were identical at 0.70 (*p* = 1.00). The sensitivity, specificity, predictive values and LHR of CRP and PCT over a range of values are presented in Tables 2 and 3. There were no adverse events reported in association with the index testing.

**Table 2:** Diagnostic test accuracy of procalcitonin for diagnosing serious infection

PCT ng/ml	Sensitivity (95% CI)	Specificity (95% CI)	Negative Predictive Value (95% CI)	Positive Predictive Value (95% CI)	Negative Likelihood Ratio (95% CI)	Positive Likelihood Ratio (95% CI)
0.25	0.80(0.44 to 0.96)	0.62(0.55 to 0.69)	0.98(0.93 to 1.00)	0.10(0.05 to 0.19)	0.32(0.09 to 1.12)	2.12(0.48 to 3.00)
0.50	0.70(0.35 to 0.92)	0.65(0.58 to 0.72)	0.98(0.93 to 1.00)	0.10(0.05 to 0.19)	0.46(0.18 to 1.19)	2.02(1.28 to 3.16)
0.75	0.60(0.27 to 0.86)	0.81(0.74 to 0.86)	0.98(0.93 to 0.99)	0.14(0.06 to 0.29)	0.49(0.23 to 1.06)	3.13(1.75 to 5.61)
1.00	0.60(0.27 to 0.86)	0.84(0.78 to 0.89)	0.98(0.93 to 0.99)	0.16(0.07 to 0.33)	0.48(0.22 to 1.02)	3.74(2.05 to 6.81)
1.25	0.60(0.27 to 0.86)	0.88(0.82 to 0.92)	0.98(0.93 to 0.99)	0.20(0.08 to 0.39)	0.46(0.21 to 0.98)	4.83(2.57 to 9.05)
1.50	0.60(0.27 to 0.86)	0.88(0.82 to 0.92)	0.98(0.93 to 0.99)	0.21(0.09 to 0.40)	0.45(0.21 to 0.97)	5.03(2.67 to 9.50)
1.75	0.50(0.20 to 0.80)	0.91(0.85 to 0.94)	0.97(0.93 to 0.99)	0.22(0.08 to 0.44)	0.55(0.30 to 1.03)	5.36(2.51 to 11.46)
2.00	0.40(0.14 to 0.73)	0.92(0.87 to 0.95)	0.97(0.93 to 0.99)	0.20(0.07 to 0.44)	0.65(0.39 to 1.09)	4.83(1.98 to 11.80)

*CI Confidence Interval*

**Table 3:** Diagnostic test accuracy of C-reactive protein for diagnosing serious infection

CRP mg/l	Sensitivity (95% CI)	Specificity (95% CI)	Negative Predictive Value (95% CI)	Positive Predictive Value (95% CI)	Negative Likelihood Ratio (95% CI)	Positive Likelihood Ratio (95% CI)
10	0.90(0.54 to 0.99)	0.40(0.33 to 0.48)	0.99(0.92 to 1.00)	0.07(0.04 to 0.14)	0.25(0.04 to 1.61)	1.51(1.19 to 1.91)
20	0.70(0.35 to 0.92)	0.52(0.45 to 0.59)	0.97(0.91 to 0.99)	0.07(0.04 to 0.14)	0.58(0.22 to 1.51)	1.45(0.94 to 2.24)
30	0.60(0.27 to 0.86)	0.63(0.55 to 0.69)	0.97(0.92 to 0.99)	0.08(0.03 to 0.17)	0.64(0.30 to 1.37)	1.61(0.94 to 2.75)
50	0.50(0.20 to 0.80)	0.78(0.72 to 0.84)	0.97(0.92 to 0.99)	0.11(0.04 to 0.24)	0.64(0.34 to 1.19)	2.30(1.17 to 4.51)
75	0.40(0.14 to 0.73)	0.87(0.81 to 0.91)	0.97(0.92 to 0.99)	0.13(0.04 to 0.32)	0.69(0.42 to 1.15)	2.97(1.28 to 6.87)
100	0.40(0.14 to 0.73)	0.93(0.89 to 0.96)	0.97(0.92 to 0.99)	0.24(0.08 to 0.50)	0.64(0.39 to 1.07)	5.94(2.36 to 14.95)
150	0.20(0.04 to 0.56)	0.97(0.94 to 0.99)	0.96(0.92 to 0.98)	0.29(0.05 to 0.70)	0.82(0.60 to 1.12)	7.72(1.70 to 35.00)
200	0.10(0.01 to 0.46)	0.99(0.96 to 1.00)	0.96(0.91 to 0.98)	0.33(0.02 to 0.87)	0.91(0.74 to 1.12)	9.65(0.95 to 97.7)

*CI Confidence Interval*

## Discussion

In this study there was no difference in the performance of PCT and CRP for the recognition of SBI amongst febrile children attending the ED. In this cohort both CRP and PCT demonstrated an AUC of 0.70 (*p* = 1.00). The findings presented here are strikingly similar to those of Verbakel et al. who reported on over 5000 children from Belgium using a similar study design [13]. In that study the rate of SBI was 4.9% and the AUC for CRP was 0.76, though they did not examine PCT [13]. These data suggest that in low prevalence settings, such as the ED, that the indiscriminate use of PCT/CRP in febrile children is of limited value.

PCT may however be useful in other settings. Specifically for use in the assessment of febrile infants under 90 days of age and in the assessment of suspected sepsis [8, 10, 12]. In these settings PCT has been shown to consistently outperform CRP as the biomarker of choice (8.10,12). Further studies to explore the use of POC PCT in these settings would be welcome.

Although the presented data are from a small cohort of patients, the study was well designed and adherent to STARD standards with prospective data collection and blinding with regards to reference standard testing. The data reflect the real-world performance of PCT and CRP when used widely in febrile children attending the ED.

This study has a number of limitations however, the study was small with a low numbers of children with SBI. Within the SBIs there was an over representation of septicaemia (four) and cellulitis (three) with an under representation of pneumonia (zero). This likely reflects the study population and that most presented with a fever and rash or other symptoms of possible meningococcal infection.

The study was also limited by the high proportion of children without sufficient blood for PCT testing (*n* = 89). The population without sufficient blood for testing was similar to that of the overall study population with four SBIs (4.5%). This suggests that the reasons for not testing were due to the challenges of paediatric phlebotomy rather than any bias based on illness severity. In addition, the majority of recruited patients were from a single site and that most were under five years of age.

The findings should be cautiously interpreted, as they are not applicable to higher SBI prevalence settings such as inpatients or intensive care and are not applicable in cases of pneumonia or in infants under three months of age.

## Conclusions

Serious bacterial infections are often difficult to differentiate from viral infections during the prodrome. It has been suggested that PCT may be superior to CRP for the diagnosis of SBIs in children [8–10]. The data presented here suggests that PCT and CRP both have only a moderate accuracy for detecting serious bacterial infections when use indiscriminately in febrile children attending the emergency department.

## Data Availability

All of the individual participant data collected during this study will be available (including data dictionaries) on the Queen’s University Belfast data repository. The full study protocol is available as an open access publication.

## Abbreviations

CRP: C-reactive protein
ED: Emergency department
LHR: Likelihood ratio
NICE: National Institute for Health and Care Excellence
NPV: Negative predictive value
OREC: Office for Research Ethics Committees
PCR: Polymerase chain reaction
PCT: Procalcitonin
PPI: Patient and public involvement
PPV: Positive predictive value
REDCAP: Research Electronic Data Capture
RWPC: Research without prior consent
SI: Serious infection
STARD: Standards for Reporting Diagnostic accuracy studies
